# Open Fracture of the Acromion: An Isolated Injury with Oblique-Type Fracture

**DOI:** 10.1155/2018/2107059

**Published:** 2018-06-10

**Authors:** Mohammad O. Alawad, Saleh Alharthi, Jameel Mahmoud, Basam Alanazi, Saad Surur

**Affiliations:** Department of Orthopaedic Surgery, King Saud Medical City, Riyadh, Saudi Arabia

## Abstract

Open acromial fractures are a rare set of fractures. We report a case of Gustilo IIIA open acromial fracture (14A2 as per OTA scapular fracture classification) that was isolated from any other injury. Our patient had a good recovery and showed excellent clinical outcome after irrigation and screw fixation of the acromial fracture. We also reviewed the literature for other cases of open acromial fracture.

## 1. Introduction

Acromial fractures are a rare entity, especially when they are open. Direct injury by a cleaver to the shoulder has caused an isolated Gustilo IIIA open acromial fracture in a young man. Because both the isolated injury and the fracture pattern were uncommon, we decided to report it.

## 2. Case Presentation

**Figure 1:**
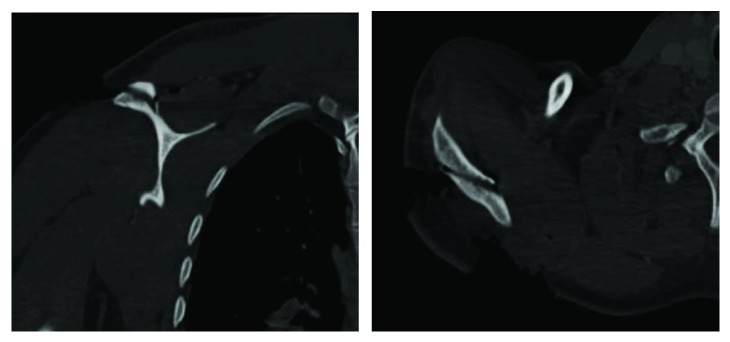
Coronal and axial computed tomography views showing the base of acromion fracture.

**Figure 2:**
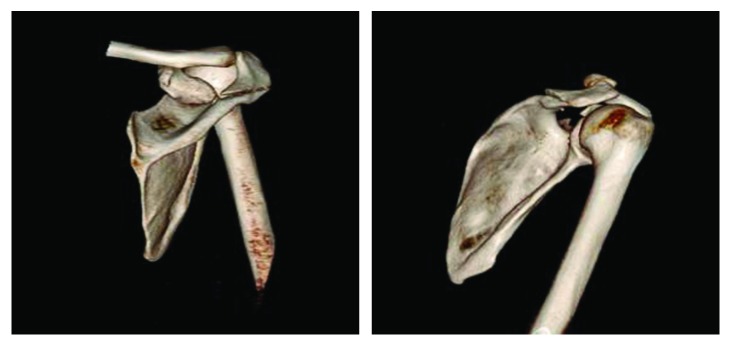
3D views showing the base of acromion fracture.

This 34-year-old male, who was medically free, was presented to the emergency department by the Red Crescent after an assault injury. He was conscious, alert, oriented, and complaining of right shoulder pain and bleeding due to assault by a cleaver. On examination, there was a wound around 20 cm on the posterior aspect of the right shoulder extending to the glenohumeral joint, acromion was exposed, and no active bleeding was present. There was no vascular or neurological injury, and passive motion and active motion of the shoulder were painful and limited. Computed tomography (CT) scan with 3D reconstruction was done prior to surgery, which confirmed a minimally displaced coronal-oblique fracture at the base of the acromion (Figures 1 and 2). Informed consent was taken from the patient to publish this case report.

## 3. Management and Outcomes

**Figure 3:**
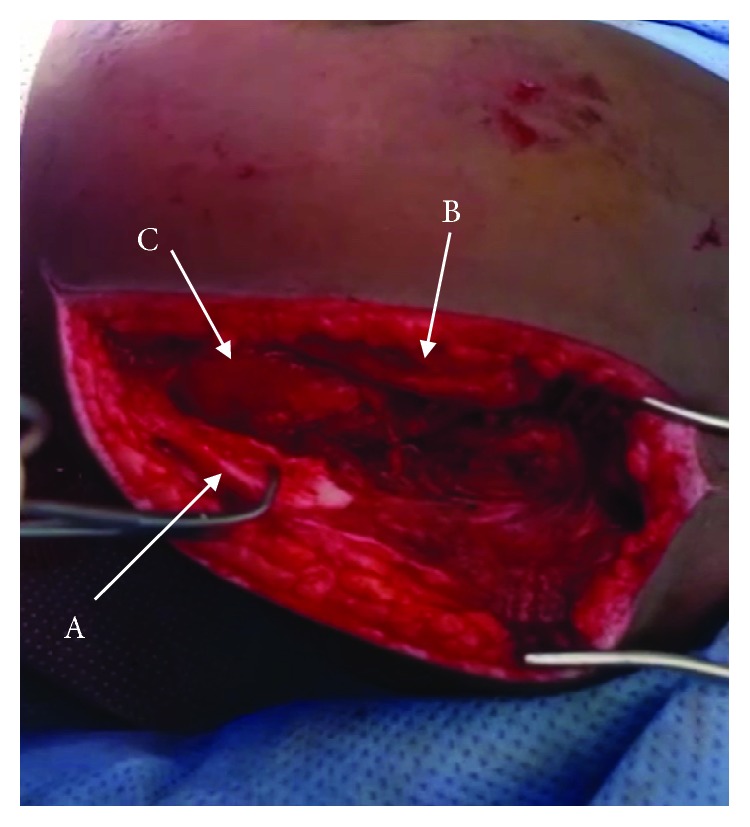
Posterolateral surgical approach showing the fractured acromion (A), scapula (B), and head of humerus (C).

**Figure 4:**
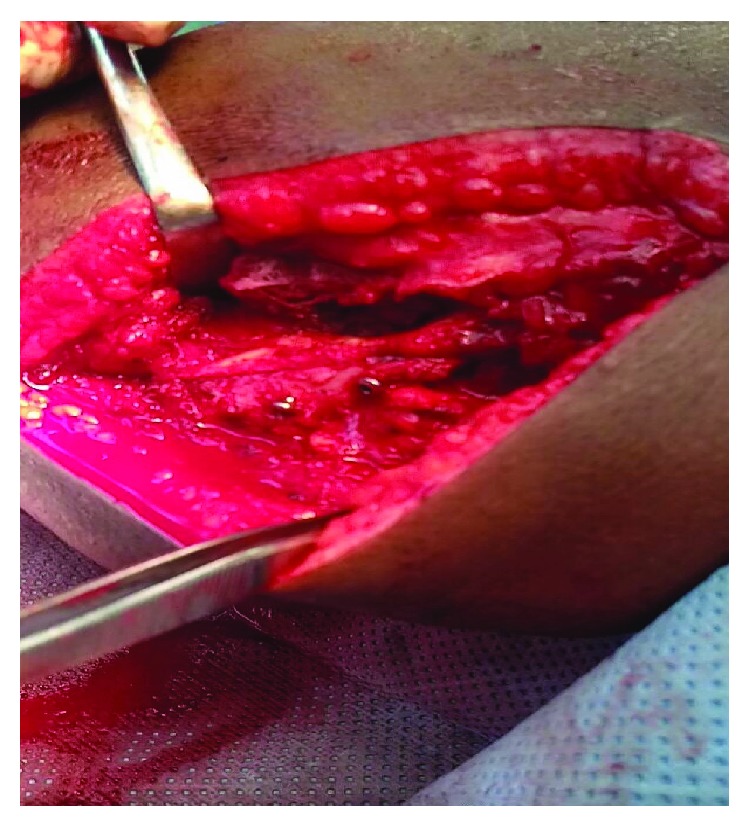
Fracture fixed with partially threaded screws.

**Figure 5:**
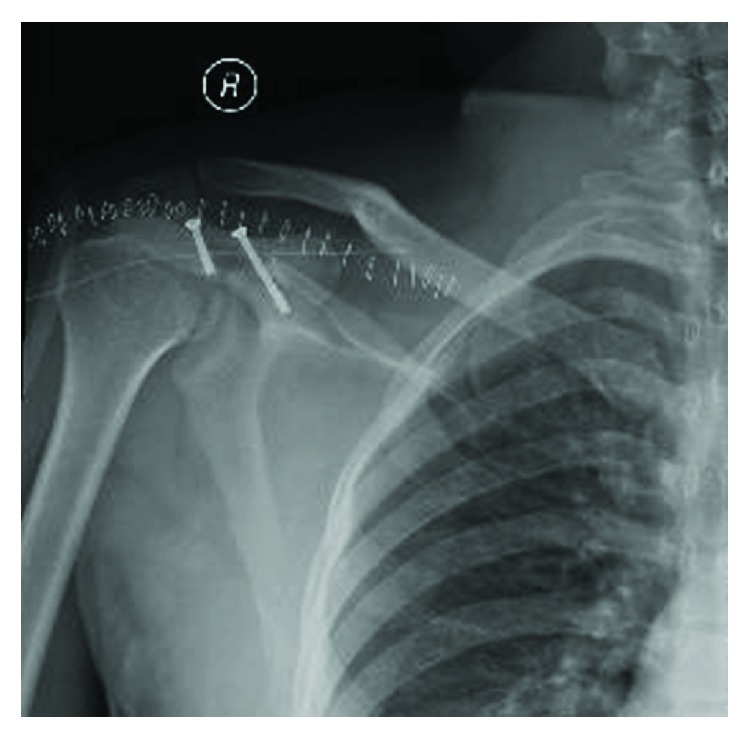
Anteroposterior X-ray of the right shoulder.

Fracture was identified as a Gustilo IIIA open fracture of the acromion [1]. Broad spectrum antibiotics were started, and patient was operated after 5 hours from the injury. Under general anesthesia and in prone position, wound was irrigated and fracture of the acromion was exposed and fixed by 2 partially threaded cannulated screws size 30 mm × 3.5 mm and 18 mm × 3.5 mm, towards the scapular spine and perpendicular to the fracture line, respectively (Figures 3–5). There was no deltoid or rotator cuff tear. Patient has then completed his antibiotic course for 72 hours and then discharged home in a good and stable condition, and the shoulder was immobilized in an arm sling for 2 weeks. At the 2-week follow-up, patient came for removal of the clips and was started on an active range of motion. There were no further hospital visits for no obvious reason.

## 4. Discussion

In general, scapular fractures are rare, comprising 1% of all fractures, and among the rarest types to happen are acromial fractures, accounting for 8-9% of all scapular fracture [2]. Therefore, the literature lacks for studies or case series showing possible concomitant injuries, complications, or evidence-based management options. We reviewed PubMed, Google Scholar, and Cochrane databases for any similar cases, which did not yield any publication with the same presentation and findings.

Given the fact that open fractures are rare, accounting for about 2.6% of all fractures [3], only one case of an open fracture of the acromion was published in 2014 [4]. In that aforementioned case report, the patient had an acromial fracture associated with supraspinatus tendon rupture. Proximity of the supraspinatus tendon to the acromion can explain the reason for the rupture.

Based on the AO/OTA Fracture and Dislocation Classification, acromion fractures are regarded as 14A2 in their alphanumeric classification system. However, fractures of coronal-oblique pattern were never discussed before in the literature.

In terms of management, the patient was treated with the best practice available for type IIIA open fractures according to Gustilo Classification: washing and good antibiotic coverage with 1st generation cephalosporins and aminoglycosides along with fracture fixation using 2 partially threaded screws for osteosynthesis [1, 5, 6].

We thought reporting this isolated open acromial fracture would contribute to the literature, especially with this fracture type, course of treatment, and excellent short-term recovery. Other case reports and series should be reported when available to show other possible conditions associated with this fracture entity.
